# Brain functional changes in patients with botulism after illegal cosmetic injections of botulinum toxin: A resting-state fMRI study

**DOI:** 10.1371/journal.pone.0207448

**Published:** 2018-11-28

**Authors:** Ge-Fei Li, Shiyu Ban, Mengxing Wang, Jilei Zhang, Haifeng Lu, Yan-Hui Shi, Xin-Wei He, Yi-Lan Wu, Peng Peng, Yi-Sheng Liu, Mei-Ting Zhuang, Rong Zhao, Xiao-Lei Shen, Qiang Li, Jian-Ren Liu, Xiaoxia Du

**Affiliations:** 1 Department of Neurology, Shanghai Ninth People's Hospital, Shanghai Jiao Tong University School of Medicine, Shanghai, China; 2 Clinical Research Center, Shanghai Jiao Tong University School of Medicine, Shanghai, China; 3 Shanghai Key Laboratory of Magnetic Resonance and Department of Physics, School of Physics and Materials Science, East China Normal University, Shanghai, China; Banner Alzheimer's Institute, UNITED STATES

## Abstract

**Background:**

Botulinum toxin type A (BoNT-A) is generally considered safe and is widely used to treat a variety of clinical conditions involving muscle hyperactivity and for cosmetic purposes. However, the effects of BoNT-A poisoning (botulism) on brain function are poorly understood.

**Methodology/Principal findings:**

Herein, we investigated brain functions in 9 patients who received illegal cosmetic injections of botulinum and 18 matched controls by combining the analysis methods of regional homogeneity (ReHo) and amplitude of low-frequency fluctuation (ALFF) based on resting-state fMRI. Compared with the controls, the patients with botulism exhibited significantly reduced ReHo values in the left posterior lobe of the cerebellum extending to the right anterior lobe of the cerebellum, as well as in the right anterior lobe of the cerebellum extending to the parahippocampal gyrus and right posterior lobe of the cerebellum. The patients with botulism also showed weakened ALFF values in the right anterior lobe of the cerebellum extending to the left anterior lobe of the cerebellum and right posterior lobe of the cerebellum, as well as in the right anterior lobe of the cerebellum.

**Conclusions/Significance:**

The results indicate that BoNT-A may modulate cerebral activation in specific areas, which may play roles in both the adverse effects of botulism and the mechanism underlying clinical treatment with BoNT-A.

## Introduction

Botulinum toxin type A (BoNT-A) is widely used to treat a variety of clinical conditions characterized by excessive muscle contraction, including dystonia and spasticity [1, 2]. BoNT-A is also used as a treatment for other medical conditions, such as neuropathic pain, axillary hyperhidrosis, neurogenic detrusor overactivity and sialorrhea [1, 3–6]. In addition, BoNT-A, alone or in combination with other agents, is exploited in a variety of cosmetic treatments. BoNT-A acts at neuromuscular junctions to inhibit the release of the neurotransmitter acetylcholine, thus weakening the contraction of muscle fibers responsible for excessive involuntary movements [1]. Both basic and clinical research has thoroughly addressed the toxin’s peripheral actions, and its mechanism of action on muscle spindles is well described [7, 8]. BoNT-A also acts on central nervous system (CNS) structures. BoNT-A may exert effects via supraspinal mechanisms that alter the balance between afferent input and motor output, thereby altering cortical excitability [9]. Furthermore, BoNT-A injected into facial muscles induces changes in the central motor organization of this body region that are independent of altered sensory input; this pattern of clinical activity is probably derived from summated peripheral and central actions [10]. Previous rodent experimental studies have shown that botulinum toxin receptors exist in the CNS and that a small amount of botulinum toxin can pass through the blood-brain barrier [10, 11].

Increasing evidence supported by neuroimaging studies in patients indicates that central reorganization occurs following BoNT-A treatment. In previous fMRI studies in patients with focal spasticity, cortical changes occurred after BoNT-A injections, and brain (such as sensorimotor and cerebellar) activity was modulated by BoNT-A [12–16]. Most previous fMRI studies in patients have investigated and described changes in cortical activity based on tasks. However, cortical changes in the resting state are unclear, and the influence of BoNT-A on brain function in healthy individuals is not yet known. We suspected that BoNT-A induces changes in brain activities not only in the task state but also in the resting state; we further postulated that the brain’s spontaneous activity would be altered by peripheral BoNT-A injection in healthy individuals.

Although BoNT-A is generally considered safe, it may cause serious adverse events after both therapeutic and cosmetic uses [17]. Adverse events associated with BoNT-A include dysarthria, dysphonia, dysphagia, respiratory compromise, generalized muscle weakness, marked bilateral ptosis, pain, bowel/bladder-related changes, blood circulation-related changes, gait-related changes, and neurological problems [17, 18]. A subject who experiences a serious adverse event after BoNT-A injection of a dose substantially exceeding that commonly used in the clinic provides an unusual opportunity to investigate the pharmacological and toxicological mechanisms of BoNT-A, especially the effect of BoNT-A on brain function. These observations could contribute to a better understanding of the therapeutic effects of BoNT-A on dystonia [1, 19], spasticity [1, 19], depression [20, 21], and pain [22, 23], among others. Moreover, a better understanding of the mechanisms underlying BoNT-A poisoning could also improve the guidelines for BoNT-A administration, as well as the outcomes, and minimize adverse events. This study aimed to investigate resting-state neural function in 9 subjects with botulism after cosmetic use of BoNT-A; we focused on the activation of motor control brain regions (such as sensorimotor and cerebellar areas), which plays an important role in BoNT-A treatment.

Herein, we analyzed resting-state fMRI data to evaluate brain function in patients with botulism and controls by applying the regional homogeneity (ReHo) and amplitude of low-frequency fluctuation (ALFF) analysis methods. The ReHo method tests for local correlations in blood oxygen level-dependent (BOLD) time series using Kendall’s coefficient of concordance (KCC) to measure regional synchronizations of temporal changes in BOLD activity [24]. ALFF changes in signals are thought to be associated with local neuronal activity, and ALFF analysis is effective at detecting fluctuations in spontaneous low-frequency oscillations. In this study, we detected changes in spontaneous brain activity by comparing ALFF and ReHo values between botulism patients and healthy controls.

## Materials and methods

The study was approved by the Independent Ethics Committee of Shanghai Ninth People’s Hospital (No. 2016-44-T1). All patients and healthy controls provided written informed consent using forms approved by the committee. All the investigations were conducted according to principles expressed in the Declaration of Helsinki.

### Subjects

Nine female patients with BoNT-A poisoning (botulism) were recruited from the Department of Neurology at Shanghai Ninth People’s Hospital of the Shanghai Jiao Tong University School of Medicine from October 2016 to December 2016. An attending neurologist diagnosed these patients as having botulism after cosmetic BoNT-A injection based on their medical history, clinical manifestations, and various supplementary examinations. Patient demographic and clinical data are shown in Table 1. The average age of the patients was 29.4±5.5 years, the average time from injection to onset of botulism symptoms was 5.4±4.1 days, the average time from onset to significant symptom relief was 35.6±11.2 days, the average time from onset to total recovery was 59.4±13.2 days, and the average time from fMRI scan to botulism onset was 39.4±14.6 days.

**Table 1:** Clinical data of patients with botulinum toxin type A poisoning.

Patient ID	Age (years)	Clinical manifestation	Injection site	Bottles*	Time from injection to symptom onset (days)	Time from onset to significant symptom relief (days)	Time from onset to total recovery (days)	Time from injection to fMRI scan (days)
1	29	Dizziness, dysphagia, fatigue, and dyspnea	Bilateral gastrocnemius muscle	1	7	28	70	57
2	43	Blurred vision and diplopia	Forehead, bilateral ocular area	2	14	30	60	64
3	33	Blurred vision, diplopia, salivation, dysarthria, dysphagia, and dyspnea	Bilateral gastrocnemius muscle	2	3	60	90	53
4	26	Dysphagia, tongue stiffness, and drooping eyelids	Bilateral gastrocnemius muscle	3	7	30	50	37
5	25	Diarrhea, blurred vision, numbness of limbs, and dyspnea	Bilateral gastrocnemius muscle	3	0.5	30	60	37.5
6	31	Dizziness, dysphagia, blurred vision, diplopia, and photophobia	Forehead, bilateral ocular area	2	5	30	55	21
7	28	Dizziness, chest tightness, fatigue, blurred vision, dysarthria and dysphagia	Bilateral masseter muscle	3	3	45	60	46
8	25	Fatigue, weakness of the neck, dysphagia, blurred vision, and dry eyes	Bilateral gastrocnemius muscle	2	7	30	50	23
9	28	Dizziness, fatigue, and drooping eyelids	Bilateral gastrocnemius muscle	1	3.5	30	50	33.5

* The number of bottles of botulinum toxin injected according to the patient’s recollection.

All patients had botulism following cosmetic injections with an unlicensed, highly concentrated botulinum preparation. The patients received the BoNT-A injections in beauty shops. Eight patients were from Shanghai city, and one was from Nanjing city. Six subjects received BoNT-A in the bilateral gastrocnemius muscle for lean legs, 2 subjects received BoNT-A in the bilateral ocular region for facial wrinkles, and one subject received BoNT-A in the bilateral masseter muscle for bilateral masseter hypertrophy treatment. The toxin was purchased through illegal channels (such as smuggling) without a license. The concentrations of unlicensed BoNT-A preparations can be 9 times higher than that normally used in the clinic according to one test report of a similar case from a local health inspection office. However, all patients had normal blood oxygen saturation levels. Although some subjects had dyspnea, their blood gas analysis results were within the normal range, even in the most severe stage. The patients underwent MRI exams after significant relief of all their symptoms (severe systemic muscle paralysis), and they had no dyspnea, anxiety, depressive symptoms, or other discomfort during the MRI scans. Patient details are provided in Table 1.

The healthy controls were recruited from the Shanghai population, and they were age- and gender-matched (1:2) to the botulism patients. Subjects who had experienced any neurological disorders or received a BoNT-A injection within the past five years were excluded. The healthy controls had a mean age of 29.4±5.5 years (same as the patient group). All subjects were right-handed and had no history of substance abuse. All neurological and psychiatric diseases were excluded based on a clinical examination and structured interviews.

### MRI acquisition

Functional and structural MRI data were acquired using a 3.0 Tesla Siemens Trio Tim system (Siemens Healthcare, Erlangen, Germany) with a 12-channel head coil. All subjects’ head movements were minimized with custom-fit foam pads. We obtained the whole-brain anatomical volume using a high-resolution, T_1_-weighted, 3-dimensional, magnetization-prepared, rapid-acquisition, gradient-echo pulse sequence. The parameters were as follows: repetition time = 2530 ms, echo time = 2.34 ms, inversion time = 1100 ms, flip angle = 7°, number of slices = 192, sagittal orientation, field of view = 256×256 mm^2^, matrix size = 256×256, and slice thickness = 1 mm, with a 50% gap. Resting-state fMRI images were acquired using a T_2_*-weighted, gradient-echo, echo-planar imaging pulse sequence with the following parameters: repetition time = 2000 ms, echo time = 30 ms, flip angle = 90°, number of slices = 33, transverse orientation, field of view = 220 × 220 mm^2^, matrix size = 64 × 64, slice thickness = 3.5 mm, 25% distance factor, with 210 volumes in total. During the fMRI scan, the subjects were instructed to relax, remain still and close their eyes.

### Resting-state fMRI data preprocessing

Resting-state fMRI data preprocessing was conducted with statistical parametric mapping software (SPM12; http://www.fil.ion.ucl.ac.uk/spm/software/spm12) and MATLAB software (MathWorks, Natick, MA) on a personal computer. For each participant, the first 10 volumes were discarded to avoid scanner instability and to allow the participants to adapt to the scanner noise. The remaining volumes were corrected for the intravolume acquisition time delay using slice-timing correction and were realigned to the first volume using a six-parameter (rigid body) spatial transformation. After these corrections, we coregistered the high-resolution, T_1_-weighted image to the mean functional image. The T_1_ images were then segmented into gray matter, white matter and deformation field images. Next, the images were spatially normalized to the standard Montreal Neurological Institute (MNI) stereotaxic space and resampled to 3 × 3 × 3 mm^3^. Spurious signals, including the time series of six head motion parameters and signals from the white matter and cerebrospinal fluid, were regressed out using a general linear model, and linear trends were removed from the fMRI data. Finally, spatial smoothing was performed on the functional images using a Gaussian filter (6 mm full width at half-maximum, FWHM).

The head motion parameters of all participants were calculated in the translational and rotational directions (i.e., x, y, z, roll, pitch, and yaw). Participants were excluded if their maximum translation was > 2 mm or if their rotation was > 2° in any direction; no patients or controls exhibited excessive movement.

### Data analysis

#### ReHo analysis

We used Data Processing and Analysis of Brain Imaging (DPABI) v2.0 to conduct ReHo analysis based on unsmoothed data [25]. A temporal bandpass filter (0.01 < f < 0.1 Hz) was applied to reduce the influences of low-frequency drift and high-frequency respiratory and cardiac noise.

An individual ReHo map was generated by calculating the concordance of the KCC of the time series of a given voxel with those of its 26 nearest neighbors [24]. To eliminate the effect of individual diversification, the ReHo value of each voxel was converted into a z-score by subtracting the mean ReHo value and dividing the standard deviation of the whole-brain ReHo map [26]. Finally, standardized ReHo maps were spatially smoothed with a 6-mm FWHM Gaussian kernel.

#### ALFF analysis

ALFF analysis was conducted on the basis of preprocessed data. For a given voxel, the time series was transformed to the frequency domain using fast Fourier transforms, and the square root of the power spectrum was calculated and averaged across 0.01–0.1 Hz. This averaged square root was referred to as the ALFF. Finally, the ALFF value of each voxel was converted into a standardized z-score by subtracting the mean ALFF value and dividing the standard deviation of the whole-brain ALFF map such that the maps could be compared across subjects [26, 27].

### Statistical analysis

The ReHo and ALFF maps for each group were obtained by one-sample *t*-tests within a brain mask. To assess the ReHo and ALFF differences across the groups, significant differences in the ReHo and ALFF maps of the 9 patients with botulism and 18 controls were compared using voxelwise two-sample *t*-tests within a brain mask. Clusterwise familywise error (FWE) corrections were applied to control for multiple comparisons of ReHo and ALFF maps, with a voxelwise uncorrected threshold of *p* < 0.001 and a clusterwise corrected threshold of *p* < 0.02 [28]. The surviving clusters were reported.

## Results

### Demographic and clinical data of botulism patients and healthy controls

The demographic and clinical data of the patients with botulism are presented in Table 1. Age was matched between the botulism patients and the controls (p = 1).

### ReHo and ALFF

**Fig 1:**
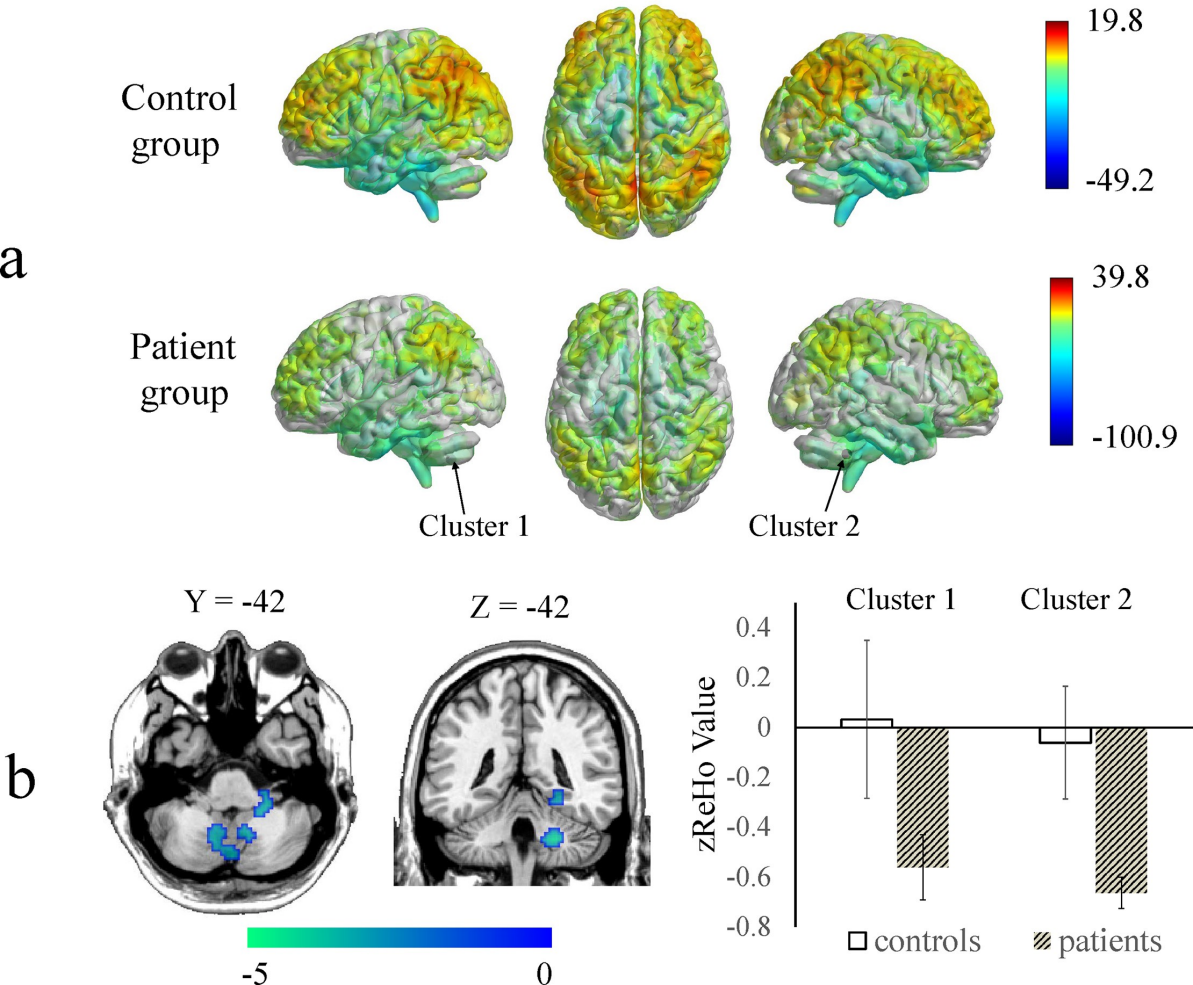
Regional homogeneity (ReHo) maps showing significant differences between the botulism patients and the healthy controls. A) ReHo maps of each group were obtained using one-sample *t*-tests (p < 0.001 and FWE-corrected to p < 0.02 at the cluster level). The dots that the arrows point to correspond to the peak positions of the clusters for the differences between two-sample *t*-tests. B) When compared with the controls, the patients exhibited significantly decreased ReHo values in the cerebellum and parahippocampal gyrus and significantly increased ReHo values in the right anterior and middle cingulate gyri. The mean ReHo z-scores within significantly altered brain areas (cluster 1 and cluster 2 in Table 2) were extracted from each subject’s data.

**Fig 2:**
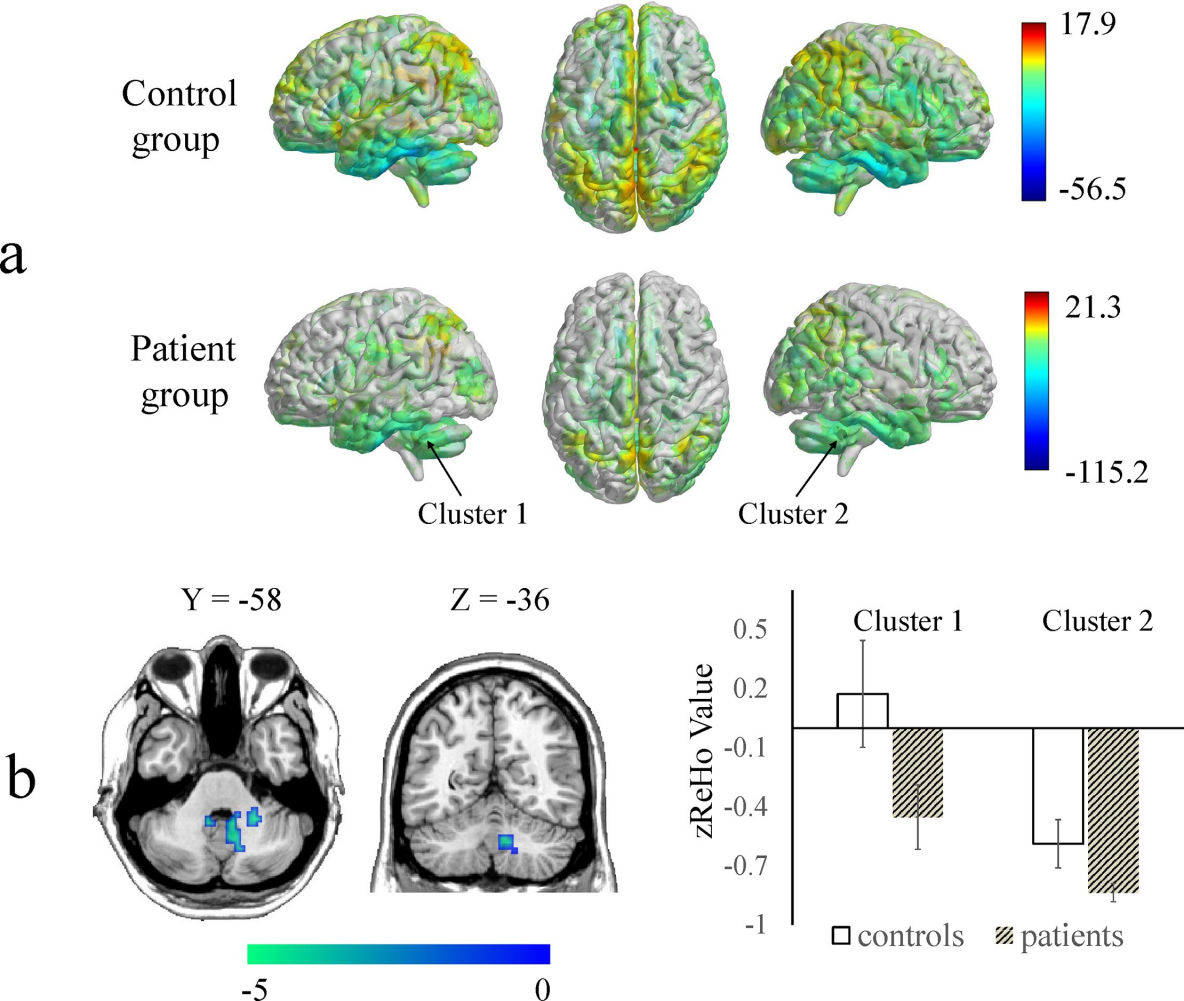
Amplitude of low-frequency fluctuation (ALFF) maps showing significant differences between the botulism patients and the healthy controls. a) ALFF maps of each group were obtained using one-sample *t*-tests (p < 0.001 and FWE-corrected to p < 0.02 at the cluster level). The dots that the arrows point to correspond to the peak positions of the clusters for the differences between two-sample *t*-tests. b) Compared with the controls, the patients exhibited significantly decreased ALFF values in the bilateral anterior lobe of the cerebellums. The mean ALFF z-scores within significantly altered brain areas (cluster 1 and cluster 2 in Table 2) were extracted from each subject’s data.

The ReHo and ALFF maps for the 9 botulism patients and 18 controls had similar distributions within groups as indicated by a one-sample *t*-test. The difference between groups was revealed by a two-sample *t*-test (Figs 1 and 2). Compared with the controls, the patients with botulism exhibited significantly decreased ReHo values in the left posterior lobe of the cerebellum extending to the right anterior lobe of the cerebellum, right anterior lobe of the cerebellum extending to the right parahippocampal gyrus and right posterior lobe of the cerebellum (Table 2 and Fig 1). Compared with the controls, the patients with botulism also showed decreased ALFF values in the right anterior lobe of the cerebellum extending to the left anterior lobe of the cerebellum and right posterior lobe of the cerebellum and right anterior lobe of the cerebellums (Table 2 and Fig 2).

**Table 2:** Significant intergroup differences in ReHo and ALFF values between patients with BoNT-A poisoning and controls.

Cluster number	Predominant regions in cluster	Cluster size	Peak *t*-value	MNI coordinates			Cluster level
				x	y	z	P_FWE-corrected_
**ReHo reduction in patients with botulism**							
Cluster 1	Left posterior lobe of the cerebellum extending to the right anterior lobe of the cerebellum	258	-6.04	-6	-63	-51	**<0.001**
Cluster 2	Right anterior lobe of the cerebellum extending to the right parahippocampal gyrus and right posterior lobe of the cerebellum	345	-5.29	21	-45	-39	**<0.001**
**ALFF reduction in patients with botulism**							
Cluster 1	Right anterior lobe of the cerebellum extending to left anterior lobe of the cerebellum and right posterior lobe of the cerebellum	87	-5.52	6	-48	-33	**< 0.001**
Cluster 2	Right anterior lobe of the cerebellum	36	-5.16	24	-45	-36	**= 0.011**

The surviving clusters were assigned thresholds at p < 0.001 and FWE-corrected to p < 0.02 at the cluster level. ReHo: regional homogeneity; ALFF: amplitude of low-frequency fluctuation; BoNT-A: botulinum toxin type A.

## Discussion

In this study, we evaluated alterations in spontaneous brain activity in patients with botulism using two different types of data-driven analyses, ReHo and ALFF. The results of the ReHo and ALFF analysis were consistent. In the patients with botulism, the ReHo and ALFF values in the cerebellar anterior and posterior lobes were decreased. In addition, the patients with botulism showed reduced ReHo values in the parahippocampal gyrus. Thus, central reorganization occurred following BoNT-A injection. The patients with botulism also showed abnormal fALFF (fractional amplitude of low-frequency fluctuation) values in the cerebellar anterior and posterior lobes and in the parahippocampal gyrus, and these findings were consistent with the ALFF and ReHo results. The details of the fALFF method and results are presented in the S1 Table.

In humans, the cerebellum is involved in processing sensorimotor, affective and cognitive information [29–31]. In addition, the anterior lobe of the cerebellum may be involved in sensorimotor function, and the posterior lobe of the cerebellum may be involved in cognitive/emotional function [29–31]. Cerebellar damage impairs both sensory and motor function [32] and produces disorders in fine movement, equilibrium, posture, and motor learning in humans [32]. The abnormal spontaneous brain activity in the cerebellum after BoNT-A injection in our study indicates that BoNT-A can modulate cerebellar (such as the anterior lobe) activity and affect both sensory and motor function. Functional MRI has been used to uncover modulation in the central reorganization associated with BoNT-A therapy for clinical conditions characterized by excessive muscle contraction. Using resting-state fMRI, Delnooz *et al*. revealed that BoNT-A treatment resulted in partial restoration of connectivity abnormalities in the sensorimotor and primary visual networks in cervical dystonia patients [33]. These findings suggest that BoNT-A directly acts on the peripheral local injection site as well as on the CNS and that BoNT-A treatment is derived from summated peripheral and central actions.

In this study, the patients with botulism showed abnormal activation in the cerebellum, consistent with previous clinical research. For example, cerebellar activation was altered after BoNT-A treatment for upper-limb spasticity [13, 16]. In one study, task fMRI was used to measure changes in cerebellar activation before and after upper-limb BoNT-A injection; cerebellar activation increased bilaterally during gripping with the paretic hand and during rest after the BoNT-A injection, suggesting that reducing spasticity with BoNT-A injection may increase cerebellar activation [16]. Veverka *et al*. investigated localized brain activation changes in stroke patients treated with BoNT-A for upper-limb spasticity using fMRI and found that BoNT-A transiently lowered modified Ashworth scale scores, and additional clusters transiently emerged bilaterally in the cerebellum, contralesional sensorimotor cortex, and contralesional occipital cortex at 4 weeks after BoNT-A injection [13]. Our results indicated that abnormal cerebellar activation may be involved in the adverse effects of BoNT-A poisoning. Conversely, our results showed that BoNT-A can modulate cerebellar (especially anterior lobe) activation, which may play an important role in the treatment of movement disorders.

We also found abnormal spontaneous brain activity in the parahippocampal gyrus after BoNT-A poisoning. How BoNT-A affects the parahippocampal gyrus remains unclear. In chronic stroke patients suffering from severe hand paralysis with arm spasticity, task-related fMRI prior to BoNT-A treatment showed extensive activation of the bilateral frontoparietal sensorimotor cortical areas, anterior cingulate gyrus, pallidum, thalamus and cerebellum [12]. The parahippocampal gyrus is part of the limbic area, and the limbic lobe and cerebellum are involved in emotion processing [29–31], which plays an important role in depression [34, 35]. Recently, published studies have shown a reduction in depressive symptoms after BoNT-A injection into the glabellar region [20, 21, 36, 37]. In our study, BoNT-A injection induced abnormal spontaneous brain activity in the cerebellum (especially the posterior lobe) and parahippocampal gyrus in patients with botulism, suggesting that BoNT-A could modulate the activity of these brain areas. These areas are involved in emotion processing and are associated with the pathophysiology of depression. Thus, further clinical studies in patients with depression are necessary to investigate the mechanism and efficacy of BoNT-A injections for the treatment of depressive disorders.

The fMRI scan was performed when the patients’ symptoms of muscle weakness had nearly disappeared; they participated in normal activities of daily living and did not have anxiety or depression because their symptoms had been significantly relieved and they knew that their symptoms were completely reversible. Therefore, we believe that mild residual muscle weakness would not have a significant impact on the fMRI results. Ultimately, we found that BoNT-A had an influence on spontaneous brain function in the subacute stage (21–64 days after injection of BoNT-A). It could be speculated that the brain function of patients would be more significantly affected in the earlier stage after injection of BoNT-A. Central effects of BoNT-A reported previously were obtained in patients with focal spasticity, and that kind of central effect could not be a direct effect of BoNT-A on the brain and must be secondary to the therapeutic effect on focal spasticity. However, we found a central effect of BoNT-A in previously healthy individuals, and our study supported the idea that BoNT-A could have a direct impact on brain function.

Our research revealed abnormal spontaneous brain activity in patients with botulism who were previously healthy, but the study had several limitations. First, the sample size of patients with botulism was small, although patients come to our hospital (the largest plastic surgery medical center in China) from all over the country. Second, this study was not a strictly designed experiment, and there were individual differences among the subjects, such as the injection position and the time from injection to fMRI scanning. However, in our study, all patients with botulism were young and previously healthy when they unexpectedly received a high dose of BoNT-A; thus, these patients offered a unique opportunity to investigate the direct effects of BoNT-A on brain function in healthy individuals. Although we demonstrated that the botulism patients showed abnormal spontaneous brain activity in the CNS, the central mechanism underlying this result was unclear. Because this study was preliminary, the results need further pharmacological and clinical validation.

## Conclusion

In this study, we determined that patients with botulism showed not only serious adverse effects on the peripheral neuromuscular junction but also abnormal spontaneous brain activity in the cerebellum and parahippocampal gyrus. Our results indicated that BoNT-A may directly modulate cerebral activation, which may be involved in both the adverse effects and the therapeutic mechanisms of BoNT-A. The findings have potential implications for the therapeutic use of botulinum toxin in complex indications in which clinical improvement cannot be explained by local muscle relaxation alone. This possibility especially applies to the emerging use of botulinum toxin in the treatment of mental disorders, such as depression.

## Supporting information

S1 Checklist Strobe checklist. (DOCX)

S1 Table Significant inter-group differences in fALFF values between patients with BoNT-A poisoning and controls. (DOCX)
