# Supplementary material for: Brain functional changes in patients with botulism after illegal cosmetic injections of botulinum toxin: A resting-state fMRI study
Source: PLoS One. 2018 Nov 28;13(11):e0207448. doi: 10.1371/journal.pone.0207448 (PMC6261580; doi:10.1371/journal.pone.0207448)
Supplement: S1 Checklist — (DOCX) [file pone.0207448.s001.docx]

STROBE Statement—checklist of items that should be included in reports of observational studies

|  | Item No. | Recommendation | Page  No. | Relevant text from manuscript |
| --- | --- | --- | --- | --- |
| **Title and abstract** | 1 | (*a*) Indicate the study’s design with a commonly used term in the title or the abstract | 1 | Tiltle：Brain functional changes in patients with botulism after illegal cosmetic injections of botulinum toxin: A resting-state fMRI study |
|  |  | (*b*) Provide in the abstract an informative and balanced summary of what was done and what was found | 2 | “In the present study, we investigated brain function in 9 patients who received illegal cosmetic injections with botulinum and 18 matched controls by combining regional homogeneity (ReHo) and amplitude of low-frequency fluctuation (ALFF) analysis methods based on resting-state fMRI.”  “Compared with the controls, the patients with botulism exhibited significantly smaller ReHo values in the left cerebellar posterior lobe and right cerebellar anterior lobe extending to the parahippocampal gyrus and stronger ReHo values in the anterior and middle cingulate gyri. The patients with botulism also showed weaker ALFF values in the left cerebellar anterior lobe, right cerebellar anterior lobe and left cerebellar posterior lobe.” |
| Introduction | | | |  |
| Background/rationale | 2 | Explain the scientific background and rationale for the investigation being reported | 3-4 | “Growing evidence supported by neuroimaging studies in patients indicates that central reorganization occurs following BoNT-A treatment. Most previous fMRI studies in patients have investigated and described changes in cortical activity based on tasks. However, cortical changes under the resting state are unclear.” |
| Objectives | 3 | State specific objectives, including any prespecified hypotheses | 4 | “We suspected that BoNT-A would induce changes in brain activities not only in the task state but also in the resting state; we further postulated that the brain’s spontaneous activity would also be altered by peripheral BoNT-A injection.” |
| Methods | | | |  |
| Study design | 4 | Present key elements of study design early in the paper | 4 | In this study, we detected changes in spontaneous brain activity by comparing ALFF and ReHo values between botulism patients and healthy controls. |
| Setting | 5 | Describe the setting, locations, and relevant dates, including periods of recruitment, exposure, follow-up, and data collection | 5-6 | Nine female patients with BoNT-A poisoning (botulism) were recruited from the Department of Neurology, Shanghai Ninth People’s Hospital, Shanghai Jiao Tong University School of Medicine from October 2016 to December 2016. These patients were diagnosed with botulism after cosmetic injection of BoNT-A by an attending neurologist based on medical history, clinical manifestation, and various supplementary examinations.  Eight patients were from Shanghai city, and one was from Nanjing city. Six subjects received BoNT-A in the bilateral crus for lower leg reduction; 2 subjects received BoNT-A in the bilateral ocular region for face-lift; and one subject received BoNT-A in the bilateral masseter muscle for face-lift. The toxin was purchased through illegal channels (such as smuggling) without a license. The concentration of the unlicensed preparation of BoNT-A could be 9 times higher than that normally used in the clinic according to one test report of a similar case from a local health inspection office. |
| Participants | 6 | (*a*) *Cohort study*—Give the eligibility criteria, and the sources and methods of selection of participants. Describe methods of follow-up  *Case-control study*—Give the eligibility criteria, and the sources and methods of case ascertainment and control selection. Give the rationale for the choice of cases and controls  *Cross-sectional study*—Give the eligibility criteria, and the sources and methods of selection of participants | 5-6 | These patients were diagnosed with botulism after cosmetic injection of BoNT-A by an attending neurologist based on medical history, clinical manifestation, and various supplementary examinations. |
|  |  | (*b*) *Cohort study*—For matched studies, give matching criteria and number of exposed and unexposed  *Case-control study*—For matched studies, give matching criteria and the number of controls per case | 6 | Eighteen age- and gender-matched healthy controls (1:2 matched with patients with botulism) that had not experienced any neurological disorders or received BoNT-A injection in the past five years were recruited. |
| Variables | 7 | Clearly define all outcomes, exposures, predictors, potential confounders, and effect modifiers. Give diagnostic criteria, if applicable | 7-8 | Participants were excluded if their maximum translation was > 2 mm or if their rotation was > 2° in any direction.  A temporal bandpass filter (0.01 < f < 0.1 Hz) was applied to reduce the influences of low-frequency drift and high-frequency respiratory and cardiac noise. |
| Data sources/ measurement | 8* | For each variable of interest, give sources of data and details of methods of assessment (measurement). Describe comparability of assessment methods if there is more than one group | 8 | The maps of the significant differences in ReHo and ALFF of the 9 patients with botulism and 18 controls were compared using voxelwise two-sample t-tests with age as a covariate. To address the issue of multiple comparisons, we assigned the ReHo and ALFF statistical map thresholds at p < 0.001 (voxel level), and family wise errors (FWE) were corrected to p < 0.05 at the cluster level. The surviving clusters were reported. |
| Bias | 9 | Describe any efforts to address potential sources of bias | 6 | Eighteen age- and gender-matched healthy controls |
| Study size | 10 | Explain how the study size was arrived at |  | not applicable |

Continued on next page

| Quantitative variables | 11 | Explain how quantitative variables were handled in the analyses. If applicable, describe which groupings were chosen and why |  | not applicable |
| --- | --- | --- | --- | --- |
| Statistical methods | 12 | (*a*) Describe all statistical methods, including those used to control for confounding | 8 | The maps of the significant differences in ReHo and ALFF of the 9 patients with botulism and 18 controls were compared using voxelwise two-sample t-tests with age as a covariate. To address the issue of multiple comparisons, we assigned the ReHo and ALFF statistical map thresholds at p < 0.001 (voxel level), and family wise errors (FWE) were corrected to p < 0.05 at the cluster level. The surviving clusters were reported. |
|  |  | (*b*) Describe any methods used to examine subgroups and interactions |  | not applicable |
|  |  | (*c*) Explain how missing data were addressed |  | not applicable |
|  |  | (*d*) *Cohort study*—If applicable, explain how loss to follow-up was addressed  *Case-control study*—If applicable, explain how matching of cases and controls was addressed  *Cross-sectional study*—If applicable, describe analytical methods taking account of sampling strategy |  | not applicable |
|  |  | (*e*) Describe any sensitivity analyses |  | not applicable |
| Results | | | | |
| Participants | 13* | (a) Report numbers of individuals at each stage of study—eg numbers potentially eligible, examined for eligibility, confirmed eligible, included in the study, completing follow-up, and analysed | 7 | Participants were excluded if their maximum translation was > 2 mm or if their rotation was > 2° in any direction; no patients or controls exhibited excessive movement. |
|  |  | (b) Give reasons for non-participation at each stage |  | not applicable |
|  |  | (c) Consider use of a flow diagram |  | not applicable |
| Descriptive data | 14* | (a) Give characteristics of study participants (eg demographic, clinical, social) and information on exposures and potential confounders |  | Table 1 |
|  |  | (b) Indicate number of participants with missing data for each variable of interest |  | not applicable |
|  |  | (c) *Cohort study*—Summarise follow-up time (eg, average and total amount) |  |  |
| Outcome data | 15* | *Cohort study*—Report numbers of outcome events or summary measures over time | 5 | The average age of the patients was 29.8±5.6 years, average time from injection to onset of botulism symptom was 5.4±4.1 days, average time from onset to significant relief of symptoms was 35.6±11.2 days, average time from onset to total recovery was 59.4±13.2 days, and average time from fMRI scan to onset of botulism was 39.4±14.6 days. |
|  |  | *Case-control study—*Report numbers in each exposure category, or summary measures of exposure |  |  |
|  |  | *Cross-sectional study—*Report numbers of outcome events or summary measures |  |  |
| Main results | 16 | (*a*) Give unadjusted estimates and, if applicable, confounder-adjusted estimates and their precision (eg, 95% confidence interval). Make clear which confounders were adjusted for and why they were included | 9 | The demographic and clinical data of the patients with botulism are presented in Table 1. Age was matched between the patients with botulism and controls (P=1). |
|  |  | (*b*) Report category boundaries when continuous variables were categorized |  |  |
|  |  | (*c*) If relevant, consider translating estimates of relative risk into absolute risk for a meaningful time period |  |  |

Continued on next page

| Other analyses | 17 | Report other analyses done—eg analyses of subgroups and interactions, and sensitivity analyses |  | not applicable |
| --- | --- | --- | --- | --- |
| Discussion | | | | |
| Key results | 18 | Summarise key results with reference to study objectives | 9 | In the patients with botulism, ReHo and ALFF values decreased in the cerebellar anterior and posterior lobes. In addition, the patients with botulism showed smaller ReHo values in the parahippocampal gyrus and stronger ReHo values in the anterior and middle cingulate gyri.  The patients with botulism also showed abnormal fALFF values in the cerebellar anterior and posterior lobes and in the parahippocampal gyrus, and these findings were consistent with the ALFF and ReHo results. The details of the fALFF method and results are presented in the S1 Table. |
| Limitations | 19 | Discuss limitations of the study, taking into account sources of potential bias or imprecision. Discuss both direction and magnitude of any potential bias | 12 | Our research revealed abnormal spontaneous brain activity in patients with botulism but had several limitations. First, the sample size of patients with botulism was small, although patients come to our hospital (the largest plastic surgery medical center in China) from all over the country. Second, this study was not a strictly designed experiment, and there are individual differences among the subjects. However, in our study, the patients with botulism were young and healthy when they unexpectedly received a high dose of BoNT-A; thus, these patients offered a unique opportunity to investigate the effect of BoNT-A on brain function. |
| Interpretation | 20 | Give a cautious overall interpretation of results considering objectives, limitations, multiplicity of analyses, results from similar studies, and other relevant evidence | 9-12 | Our results indicated that BoNT-A may modulate cerebral activation, which may be involved in both the adverse effect of BoNT-A poisoning and the mechanism of clinical treatment. |
| Generalisability | 21 | Discuss the generalisability (external validity) of the study results | 12-13 | The findings have potentially high implications for the therapeutic use of botulinum toxin in complex indications in which clinical improvement can hardly be explained by local muscle relaxation alone. This applies particularly for the emerging use of botulinum toxin in the treatment of mental disorders like depression. |
| Other information | |  | | |
| Funding | 22 | Give the source of funding and the role of the funders for the present study and, if applicable, for the original study on which the present article is based |  | This research was supported by grants from the National Natural Science Foundation of China (Nos. 81571658 to X.X. Du), National Natural Science Foundation of China (No. 81271302 to J.R. Liu), research innovation project from Shanghai Municipal Science and Technology Commission (No. 14JC1404300, to J.R. Liu), "Prevention and Control of Chronic Diseases Project" of Shanghai Hospital Development Center (No. SHDC12015310, to J.R. Liu), project from SHSMU-ION Research Center for Brain Disorders (No. 2015NKX006, to J.R. Liu), project from Shanghai Municipal Education Commission—Gaofeng Clinical Medicine Grant Support (No. 20161422 to J.R. Liu), Clinical Research Project from Shanghai Jiao Tong University School of Medicine (No. DLY201614 to J.R. Liu), and Biomedicine Key program from Shanghai Municipal Science and Technology Commission (No. 16411953100 to J.R. Liu).  The funders had no role in study design, data collection and analysis, decision to publish, or preparation of the manuscript.  The authors declare that they have no conﬂict of interest. |

*Give information separately for cases and controls in case-control studies and, if applicable, for exposed and unexposed groups in cohort and cross-sectional studies.

**Note:** An Explanation and Elaboration article discusses each checklist item and gives methodological background and published examples of transparent reporting. The STROBE checklist is best used in conjunction with this article (freely available on the Web sites of PLoS Medicine at http://www.plosmedicine.org/, Annals of Internal Medicine at http://www.annals.org/, and Epidemiology at http://www.epidem.com/). Information on the STROBE Initiative is available at www.strobe-statement.org.
