# Supplementary material for: Brain functional changes in patients with botulism after illegal cosmetic injections of botulinum toxin: A resting-state fMRI study
Source: PLoS One. 2018 Nov 28;13(11):e0207448. doi: 10.1371/journal.pone.0207448 (PMC6261580; doi:10.1371/journal.pone.0207448)
Supplement: S1 Table — (DOCX) [file pone.0207448.s002.docx]

**Supporting information**

**Methods**

**fALFF (fractional amplitude of low-frequency fluctuation) analysis**

fALFF analysis also was based on preprocessed data. To acquire the power spectrum, the time series was transformed to a frequency domain using fast Fourier transform. Then, the power spectrum was square-rooted and averaged across 0.01–0.1 Hz at each voxel. This averaged square root was viewed as the ALFF. A ratio of the amplitude averaged across 0.01–0.1 Hz to that of the entire frequency range (0–0.25 Hz) was computed at each voxel to obtain the fALFF, creating an amplitude map for the whole brain. Finally, the fALFF value of each voxel was converted into a standardized z-score by subtracting the mean fALFF value and dividing the standard deviation of the whole-brain fALFF map such that the maps could be compared across subjects.

**Statistical analysis**

The fALFF maps for each group were obtained by one-sample *t*-tests within a brain mask. To assess fALFF differences across the groups, significant differences in the fALFF maps of the 9 patients with botulism and 18 controls were compared using voxel-wise two-sample *t*-tests within a brain mask. For multiple comparisons, we assigned the fALFF statistical map thresholds to p < 0.001 (voxel level), and family wise errors (FWE) were corrected to p < 0.05 at the cluster level. The surviving clusters were reported.

**fALLF Results**

Compared with the controls, the patients with botulism also showed great fALFF values in the left cerebellum posterior lobe and smaller fALFF values in the left Pons extending to left cerebellum anterior lobe and right parahippocampa gyrus, and in the right midbrain (Stable 1).

**S1 Table. Significant inter-group differences in fALFF values between patients with BoNT-A poisoning and controls.**

| Predominant regions in cluster | Cluster  size | Peak t-value | MNI coordinates | | | Cluster level |
| --- | --- | --- | --- | --- | --- | --- |
|  |  |  | x | y | z | P_FWE-corrected_ |
| **fALFF increased in patients with botulism** | | | | | | |
| Left cerebellum posterior lobe | 31 | 5.53 | -12 | -90 | -36 | =0.009 |
| **fALFF reduction in patients with botulism** | | | | | | |
| Left Pons extending to | 415 | -6.74 | -6 | -33 | -30 | <0.001 |
| left cerebellum anterior lobe |  | -6.62 | -6 | -30 | 0 |  |
| right parahippocampa gyrus |  | -5.74 | 18 | -30 | -9 |  |
| Right cerebrum/sub-lobar | 36 | -5.52 | 3 | -9 | -6 | =0.003 |
| midbrain |  | -4.77 | 6 | -18 | -6 |  |

The surviving clusters were assigned thresholds at p < 0.001 and FWE-corrected to p < 0.05 at the cluster level. fALFF: fractional amplitude of low-frequency fluctuation; BoNT-A: botulinum toxin type A.
